# MicroRNA Expression Profiles as Biomarkers of Response to Disease-Modifying Therapies in Multiple Sclerosis: A Systematic Review

**DOI:** 10.3390/ijms27146138

**Published:** 2026-07-09

**Authors:** Mihai-Ioan Dumitreasă, Smaranda Maier, Laura Bărcuțean, Doina Manu, George-Andrei Crauciuc, Otilia Buțiu, Rodica Bălașa

**Affiliations:** 1 Doctoral School, George Emil Palade University of Medicine, Pharmacy, Science, and Technology of Targu Mures, 540142 Târgu Mureș, Romania; 2 Neurology Department, George Emil Palade University of Medicine, Pharmacy, Science, and Technology of Targu Mures, 540142 Târgu Mureș, Romania; 3 Center for Advanced Medical and Pharmaceutical Research, George Emil Palade University of Medicine, Pharmacy, Science, and Technology of Targu Mures, Gheorghe Marinescu 38, 540139 Târgu Mureș, Romania; 4 Department of Psychiatry, Faculty of Medicine, George Emil Palade University of Medicine, Pharmacy, Science and Technology of Targu Mures, 540142 Târgu Mureș, Romania

**Keywords:** multiple sclerosis, microRNAs, biomarkers, treatment response, disease-modifying therapies

## Abstract

Although microRNAs (miRNAs) are an active area of research in multiple sclerosis (MS) and have been proposed as potential biomarkers of treatment response, the evidence remains difficult to interpret. This systematic review examines the relationship between miRNA expression and response to disease-modifying therapies (DMTs) in adults with MS. The PubMed/MEDLINE and Web of Science databases were systematically searched from inception to 11 February 2026. Fifteen studies that compared miRNA expression between responders and non-responders or assessed changes in miRNA expression after DMT initiation were synthesized narratively by treatment group and outcome, in accordance with the 2020 PRISMA guidelines. After considering study design, treatment response definitions, and miRNA analytical methods, miR-548a-3p in fingolimod-treated patients and miR-223-3p, miR-23a/b-3p, and miR-27a/b-3p in dimethyl fumarate (DMF)–treated patients appeared the most promising miRNAs investigated; however, each was assessed in a single study and has not yet been validated in independent external cohorts. However, they were notable because they had been evaluated in clinically relevant settings: miR-548a-3p in relation to no evidence of disease activity–3 and receiver operating characteristic-based discrimination, and the DMF-associated miRNAs through baseline expression levels and early fold-change analyses. Although miR-23a-3p, miR-26a-5p, miR-146a-5p, miR-155, miR-34a-5p, miR-223-3p, miR-660-5p, and miR-326 had been reported in more than one study, most were investigated in different DMT or outcome contexts. Therefore, the existing literature correlating miRNA expression levels with treatment response provides valuable but limited and heterogeneous evidence. Thus, miRNAs should currently be considered exploratory biomarkers and require further independent validation before their use in clinical practice.

## 1. Introduction

Multiple sclerosis (MS) is a chronic, heterogeneous immune-mediated disease of the central nervous system (CNS). It is one of the main causes of long-term neurological disability in young adults, imposing a substantial burden on their quality of life [1].

The pathogenesis of MS involves complex interactions between genetic and environmental factors, which lead to dysregulation of both the innate and adaptive immune systems [2]. In the periphery, B cells contribute to the differentiation of T helper cells into distinct subsets, among which T helper 1 (Th1) and 17 (Th17) are the most relevant in the pathogenesis of MS. These cells promote disruption of the blood–brain barrier (BBB) and subsequently infiltrate the CNS [3]. They initiate a complex chain of immune reactions that also involve resident microglia and astrocytes, leading to demyelination and axonal and neuronal damage. Glial cells compartmentalize the pathological process by forming follicle-like structures that support chronic immune activation [4].

The remarkable progress in understanding the underlying pathogenic mechanisms has led to the development of disease-modifying therapies (DMTs) that aim to reduce inflammatory activity, lower relapse rates, and slow disease progression. However, the variability in treatment response represents a major challenge in clinical decision-making and highlights the need for reliable tools to evaluate, monitor, and predict treatment response [5]. Although relapse rate, disability progression, and lesional activity on magnetic resonance imaging (MRI) are commonly used clinical and radiological measures for assessing therapeutic outcomes, they provide limited insight into the underlying molecular and immunological mechanisms of MS [6]. Neurofilaments (NfLs), which reflect axonal damage, and glial fibrillary acidic protein (GFAP), which is mainly expressed in the CNS by mature astrocytes, have emerged as potential serum and cerebrospinal fluid (CSF) biomarkers of MS progression. However, they lack disease specificity, as their levels also increase in other neurodegenerative disorders [7,8].

MicroRNAs (miRNAs) are small, non-coding ribonucleic acid (RNA) molecules that modulate gene expression in the post-transcriptional phase by binding to specific messenger RNAs, leading to their degradation or inhibition of their translation into proteins [9]. Unlike NfLs and GFAP, which become elevated as a response to tissue injury, miRNAs seem to be involved in the early phases of the pathogenesis of MS by regulating the differentiation, maturation, and function of T and B cells, as well as glial cells [9,10]. miRNAs can be isolated from various body fluids, including plasma and CSF, as well as exosomes, which can indicate their cellular origin [11]. Several studies have investigated the dynamics of certain miRNAs, showing not only that their expression is dysregulated in patients with MS compared to healthy controls, but also that their expression is influenced by different DMTs [12,13,14,15]. These findings suggest that miRNAs may serve as promising biomarkers for diagnosing, assessing treatment response, and monitoring progression of MS, as well as potential therapeutic targets [11,16,17]. Moreover, the role of miRNAs as biomarkers of MS progression and transition from relapsing–remitting MS (RRMS) to secondary progressive MS (SPMS) has been extensively studied [18,19,20].

Despite increasing research on miRNA profiles in MS, limitations such as small sample sizes and heterogeneity in the examined miRNAs, study designs, and patient populations persist and prevent the validation of clinically applicable biomarkers. Therefore, this systematic review aims to evaluate the current evidence on the relationship between miRNA expression and response to DMTs in adult patients with MS, investigating differences between responders and non-responders and longitudinal changes before and after treatment initiation.

## 2. Materials and Methods

### 2.1. Objectives

This study aimed to systematically review the evidence on blood- and CSF-derived miRNA expression profiles, including exosomal miRNAs, as biomarkers of response to DMTs in adults with MS, and to evaluate treatment-related longitudinal changes in miRNA expression in relation to treatment response.

### 2.2. Protocol Registration

This systematic review was conducted and reported in accordance with the 2020 Preferred Reporting Items for Systematic reviews and Meta-Analyses (PRISMA) guidelines. Its protocol was pre-registered in the international systematic review registry, PROSPERO (registration number: CRD420261305887, available in the Supplementary Materials).

### 2.3. Eligibility Criteria

Studies were considered eligible for inclusion if they met the following predefined eligibility criteria, structured according to the PICOS (Population, Intervention, Comparison, Outcome, Study Design) framework.

#### 2.3.1. Population

Adults aged 18 years or older diagnosed with RRMS according to the McDonald criteria or other established diagnostic criteria. Studies including mixed MS phenotypes (RRMS, SPMS, or primary progressive MS) were considered only if data for patients with RRMS were reported separately or if most participants had RRMS.

#### 2.3.2. Intervention

Treatment with approved DMTs.

#### 2.3.3. Comparison

Comparison of miRNA expression between treatment responders and non-responders, or changes in miRNA expression following initiation of a DMT, only if evaluated in relation to clinical or radiological treatment response.

#### 2.3.4. Outcome

Primary outcome: Association between miRNA expression levels and treatment response, as defined by clinical and/or radiological criteria (e.g., relapse rate, disability progression, or lesional activity on MRI).

Secondary outcome: Longitudinal changes in miRNA expression following initiation of DMTs, when evaluated in relation to clinical and/or radiological treatment response.

#### 2.3.5. Study Design

Observational studies (prospective or retrospective), including longitudinal and cross-sectional designs, were eligible. Studies assessing differences in miRNA expression between treatment responders and non-responders and/or longitudinal changes before and after treatment initiation were included. In vitro studies, animal studies, case reports, reviews, editorials, and conference abstracts without full text were excluded.

#### 2.3.6. Sample Type

Studies evaluating miRNA expression in plasma, serum, whole blood, peripheral blood mononuclear cells (PBMCs), peripheral blood leukocytes (PBLs), exosomes, or CSF were eligible.

#### 2.3.7. Language and Publication Date

Only studies published in English were eligible. No date restrictions were applied.

### 2.4. Study Identification

The PubMed/MEDLINE and Web of Science Core Collection databases were systematically searched from inception to 11 February 2026. The search strategy combined controlled vocabulary (medical subject headings [MeSH]) and free-text terms related to MS, miRNAs, and DMTs (interferon-beta [IFN-β], glatiramer acetate [GA], dimethyl fumarate [DMF], teriflunomide, fingolimod (formerly FTY720), siponimod, ozanimod, ponesimod, sphingosine 1 phosphate receptor modulators, natalizumab, ocrelizumab, ofatumumab, cladribine, alemtuzumab, ublituximab, mitoxantrone, and diroximel fumarate). The complete search strategy for both databases is provided in the Supplementary Materials.

To identify further relevant studies, backward and forward citation tracking of all included articles was performed using Web of Science. Additionally, a supplementary search of Google Scholar was performed to identify potentially missed studies. The first 100 results sorted by relevance were screened using simplified keyword combinations for MS, miRNAs, and treatment response.

### 2.5. Study Selection

Study selection was performed in two stages. First, all retrieved records were exported to EndNote v 20.2.1 (Clarivate, Philadelphia, PA, USA), and duplicates were removed. The remaining records were then imported into the Rayyan platform (Rayyan, Cambridge, MA, USA) for screening. Two reviewers independently screened the titles and abstracts for relevance according to the predefined PICOS criteria. Second, the same reviewers independently assessed the full texts of articles reporting potentially eligible studies, and any discrepancies were resolved through discussion until consensus was reached.

Studies were included if they evaluated the association between miRNA expression and treatment response in adults with RRMS receiving approved DMTs. Treatment response was assessed based on clinical or radiological outcomes, or composite outcomes, specifically no evidence of disease activity–3 (NEDA-3) status: no clinical relapses, no confirmed disability progression (CDP), and no new/enlarging lesions on MRI.

Studies were excluded if they did not evaluate treatment response using defined clinical or radiological criteria (e.g., responders vs. non-responders) or if they analyzed miRNAs only in relation to other predictive biomarkers without clinical or radiological response assessment.

### 2.6. Data Extraction

Data were extracted using a predefined data extraction form developed by the authors. The extracted information included study characteristics (first author, year of publication, country of origin, and study design) and participant characteristics (number of included patients at each time point, MS phenotype, diagnostic criteria used, mean age, mean Expanded Disability Status Scale [EDSS], MS duration, and sex distribution). Type of DMT, treatment duration, number of treatment-naïve and previously treated patients, washout period (if reported), follow-up duration, and the definition of treatment response were also recorded. Other information regarding sample type, miRNA profiling methods, RNA extraction kit, normalization strategies, and the specific miRNAs analyzed was also recorded. For each study, the direction of miRNA expression change (upregulated or downregulated), the statistical significance, the statistical test, and the reported associations between miRNA expression levels and treatment response were recorded.

### 2.7. Risk of Bias Assessment

The risk of bias was assessed using the cohort version of the Newcastle–Ottawa Scale (NOS) for prospective longitudinal studies, the case–control version of the NOS for studies comparing treatment responders and non-responders, and the JBI 2017 Critical Appraisal Checklist for Analytical Cross-Sectional Studies for cross-sectional studies. For case–control studies, non-responders/“poor” responders were considered cases, and responders/“good” responders were considered controls. Healthy controls or untreated MS groups, when present, were considered auxiliary comparator groups and were not used as controls for assessing the risk of bias in treatment response. In the cohort version of the NOS, the item “selection of the non-exposed cohort” was scored only when studies included a separate comparator cohort defined by exposure status. For these studies, responder/non-responder grouping was considered an outcome stratification rather than an exposure status. Healthy control groups or untreated individuals, when present, were not considered appropriate non-exposed cohorts because they were not part of the treated MS population in which treatment response was evaluated. In the cohort version of the NOS, the “exposure” item was considered treatment with DMTs, whereas in the JBI 2017 Critical Appraisal Checklist, “exposure” refers to biomarker (miRNA) expression level.

### 2.8. Data Synthesis

The findings of the included studies were narratively synthesized. Due to substantial heterogeneity in study design, definitions of treatment response, analyzed miRNAs, miRNA profiling methods, and biological sample types, a quantitative meta-analysis was not feasible. Study characteristics and main findings were first summarized descriptively in tabular form, including information on patient population, DMT, biological sample, miRNA profiling method, definition of treatment response, and key results.

Then, the primary and secondary outcomes were synthesized narratively, grouping studies by the evaluated DMT. Where possible, miRNAs investigated in more than one study were examined for consistency in direction and strength of association across studies. Findings were interpreted qualitatively, with particular attention to methodological heterogeneity, sample type, analytical platform, and outcome definitions that could influence comparisons between studies.

## 3. Results

### 3.1. Study Selection

**Figure 1:**
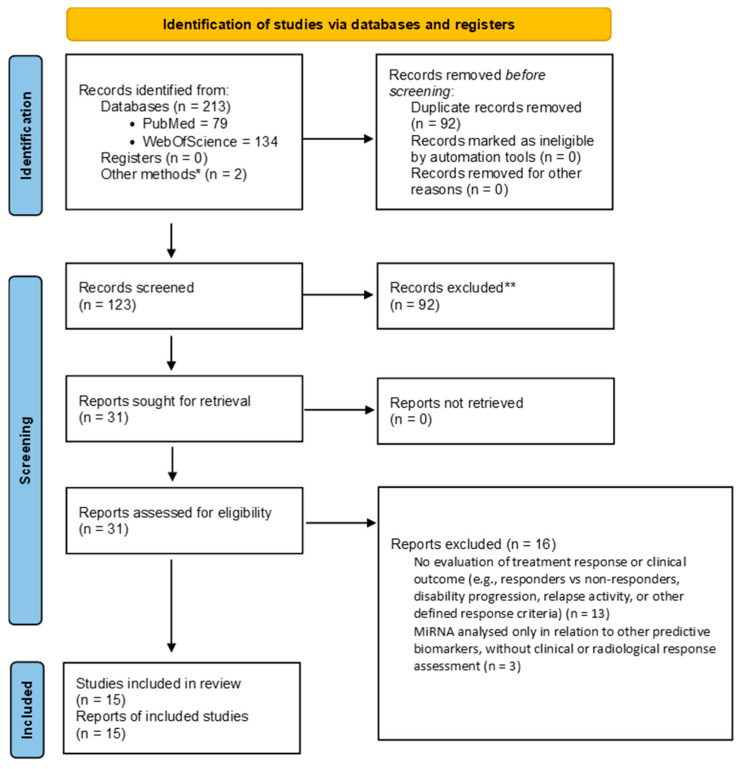
PRISMA flow diagram of study selection. * Backward and forward citation search. ** All records were excluded manually.

The study identification and selection process is summarized as a PRISMA flow diagram (Figure 1). The literature searches identified 213 records. After removing duplicates, 123 records were screened based on their titles and abstracts, of which 31 then underwent full-text review. Ultimately, 15 studies met the eligibility criteria and were included in the qualitative synthesis. No additional eligible studies were identified through forward and backward citation tracking. Although two additional records were identified through forward citation tracking, they were excluded following title and abstract screening. No additional eligible studies were identified through the supplementary search of Google Scholar.

### 3.2. Characteristics of the Included Studies

#### 3.2.1. Study Design

Among the 15 included studies, seven (46.7%) employed a prospective longitudinal observational design [13,21,22,23,24,25,26], and their analytical approaches and clinical follow-up durations are summarized in Table 1. They obtained miRNA samples and evaluated patients at multiple time points over a follow-up period of 6 months to 5 years. Only two of these studies determined miRNA expression at a single time point [13,22]. These studies primarily assessed longitudinal changes in miRNA expression following treatment initiation, either within responder groups at baseline and predefined follow-up time points or through comparisons between responders and non-responders. 

Three studies (20.0%) employed an observational case–control design and defined treatment response retrospectively after follow-up periods of 6 months to 2 years [27,28,29]. Their analytical approaches and clinical follow-up durations are presented in Table 2. In these studies, miRNA expression was primarily compared between responders and non-responders. Two studies additionally compared patients with MS and healthy controls [27,29]. In contrast, Magner et al. compared treated patients classified as “good” or “poor” responders with untreated patients and also evaluated miRNA expression before and 48 h after IFN-β injection [28].

Five studies (33.3%) employed a cross-sectional design and defined treatment response retrospectively after a follow-up period of 1 to 2 years [15,30,31,32,33]. Their analytical approaches and clinical follow-up durations are presented in Table 3. In these studies, miRNA samples were obtained at a single time point, at the end of the follow-up period, and miRNA expression was compared between treatment responders and non-responders. However, one study differed in design, comparing treated patients (responders/non-responders) with treatment-naïve patients [31].

Altogether, the 15 included studies captured different temporal relationships between miRNA expression and treatment response, which influenced whether candidate miRNAs could be interpreted as predictive biomarkers, longitudinal monitoring biomarkers, or biomarkers reflecting treatment response.

#### 3.2.2. Study Population and DMT Characteristics

Patients’ characteristics are summarized in Table 4, and their treatment exposure and prior treatment characteristics are presented in Table 5. The 15 included studies were published between 2014 and 2025 and investigated adult patients with RRMS. One also included patients with SPMS, but most participants had RRMS [28]. Regarding the criteria used to diagnose MS, 11 studies (73.3%) used the McDonald criteria, while 2 (13.3%) used the Poser criteria [27,29], and 2 (13.3%) did not report the diagnostic criteria used [22,28].

Six studies (40.0%) enrolled treatment-naïve patients [21,23,24,25,31], whereas seven (46.7%) included only patients who had previously undergone specific DMTs [15,22,28,29,30,32,33]. Two (13.3%) did not specifically report participants’ prior treatment status [26,27]. Only Giuliani et al. reported a washout period before treatment initiation [25].

IFN-β was the most frequently investigated DMT (n = 7) [15,23,28,30,31,32,33], followed by fingolimod (n = 4) [13,24,27,29], DMF (n = 2) [21,25], and GA (n = 1) [22]. Notably, only Torres-Iglesias et al. and Magner et al. investigated multiple DMTs within the same cohort [26].

Most of the included studies had relatively small sample sizes, ranging from 11 patients receiving IFN-β in Manna et al. [31] to 105 patients treated with IFN-β in Tahmasebivand et al. [33].

Overall, the available evidence focused on a limited number of DMTs, particularly IFN-β, fingolimod, and DMF. Therefore, the miRNAs proposed as potential treatment-response biomarkers should be interpreted within the specific DMT context in which they were investigated. For several treatment groups, the limited number of available studies restricts the strength of the conclusions that can be drawn.

#### 3.2.3. Definition of Therapeutic Response

The definitions of treatment response varied across the 15 included studies, and they are detailed in Table 6. Three studies (20.0%) defined treatment response based on the achievement of NEDA-3 [13,22,26], while one also used achievement of NEDA-4 (NEDA-3 plus no cognitive progression) and NEDA-5 (NEDA-4 plus brain atrophy ≤ 0.4% in the past 12 months) [26]. One study separated “stable responders” from “positive responders” based on lesion activity on MRI (gadolinium [Gd]-enhancing lesions at baseline and 6 months). Another study determined responder status (“good” vs. “poor”) solely on EDSS progression [21], whereas 10 studies used combinations of EDSS progression, relapse occurrence, and lesion activity on MRI [15,23,25,27,28,29,30,31,32,33].

Overall, the definitions of treatment response captured different aspects of disease control. The miRNA findings were reported in two main contexts: global treatment benefit and specific components of disease activity. Therefore, the miRNAs reported in relation to composite outcomes may be more informative for overall treatment benefit, whereas those reported in relation to isolated outcomes may be more relevant to specific clinical or radiological aspects of disease activity.

#### 3.2.4. Sample Types and Analytical Methods

The sample types and analytical methods employed across the 15 included studies are summarized in Table 7. Overall, miRNAs were isolated from various biological sample types, including serum/plasma, PBMCs/PBLs, peripheral whole blood, and exosomes.

Nine studies (60.0%) collected samples for miRNA isolation at a single time point [13,15,22,27,29,30,31,32,33], whereas six (40.0%) collected samples at baseline and at predefined time intervals [21,23,24,25,26,28]. Ten studies (66.7%) quantified miRNA expression using reverse transcription–quantitative polymerase chain reaction (RT-qPCR) methods, including SYBR Green or TaqMan assays [13,15,21,22,25,27,29,30,32,33], whereas four (26.7%) used high-throughput methods, including small RNA sequencing or small RNA cloning and sequencing [23,24,26,28]. In contrast, three studies (20.0%) used broader analytical methods followed by RT-qPCR validation of selected miRNAs [13,23,31]. Notably, different RNA extraction kits and protocols were used across studies. The determined miRNA levels were normalized using endogenous controls, spike-in controls, global mean normalization, or sequencing-specific normalization methods across studies.

Overall, the differences in biological compartments and analytical approaches should be considered, as circulating, cellular, and extracellular vesicle-derived miRNAs may not provide equivalent biological information. Differences in profiling platforms, normalization methods, and analytical thresholds may influence which miRNAs are detected and reported as treatment-response candidates.

### 3.3. Primary Outcome: Association Between miRNA Expression and Treatment Response

#### 3.3.1. Overview of the Findings

Nine studies (60.0%) met the criterion for the primary outcome and evaluated the association between miRNA expression and treatment response by comparing responders and non-responders according to predefined response criteria [13,15,21,26,27,29,30,32,33]. Most of these studies measured miRNA expression at a single post-treatment time point, whereas only Carbone et al. and Torres-Iglesias et al. included sampling at baseline. Carbone et al. was the only study that also evaluated treatment-induced fold changes in miRNA expression between responder groups [21].

Two studies, which used alternative designs, including baseline prediction of outcomes and cross-sectional comparisons between treated and treatment-naïve patients, were considered in this section [22,31]. Although these studies do not directly compare responders and non-responders, they were included as they provide additional evidence on the relationship between miRNA expression and treatment-related outcomes.

Across the primary outcome analyses, five miRNAs were reported in more than one study: miR-23a-3p, miR-146a-5p, miR-223-3p, miR-660-5p, and miR-326. However, these miRNAs were evaluated in different DMT contexts, and none was consistently replicated within the same treatment group. Because various DMTs had been investigated, the evidence was synthesized according to treatment group.

#### 3.3.2. Interferon-Based Therapies

Four studies (26.7%) evaluated IFN-β in relation to the primary outcome [15,30,32,33], and their details are summarized in Table 8. Notably, two studies (Fattahi et al. 2019 and Fattahi et al. 2020) were conducted by the same research group and in similar clinical settings. Thus, they may include overlapping patient cohorts, although this was not explicitly stated. The same issue was noted in the studies by Mousavi et al. and Tahmasebivand et al.

All of these studies were cross-sectional and quantified miRNA expression by RT-qPCR at a single time point after 1 year of treatment, the period used to define responders based on combinations of EDSS, relapse, and lesion activity on MRI. These studies compared miRNA expression between responders and non-responders and identified several miRNAs that were significantly dysregulated across groups. For instance, miR-29b-3p, miR-29-5p, and miR-185-5p were significantly downregulated in responders compared to non-responders [15,32], while miR-504 was significantly upregulated [33]. However, Fattahi et al. found no significant differences in the expression of the investigated miRNAs between responders and non-responders [30]. Thus, despite similarities in study design, findings varied across studies, and no consistent miRNA was identified. In addition, Manna et al. reported significant upregulation of miR-22-3p and miR-660-5p in treated patients compared to treatment-naïve patients, with modulations observed in responders [31].

In summary, the response to IFN-β was associated with downregulation of miR-29b-3p, miR-29b-5p, and miR-185-5p but with upregulation of miR-504 expression in responders. Although these miRNAs emerged as potential markers of the response to IFN-β, each was supported by only a single study, and none were consistently replicated across independent IFN-β cohorts.

#### 3.3.3. Fingolimod

Three studies (20.0%) investigated associations between miRNA expression and response to fingolimod treatment [13,27,29], and their details are summarized in Table 9. Among them, two case–control studies were conducted by the same research group and appear to include similar patient cohorts [27,29]. They quantified miRNA expression by RT-qPCR in peripheral whole blood at a single time point after treatment initiation, comparing responders and non-responders, as well as healthy controls and different responder subgroups. Notably, they found no significant differences in miRNA expression between responders and non-responders. However, Mazdeh et al. reported higher miR-381-3p expression in responders than in healthy controls and lower miR-655-3p expression in responders and non-responders than in healthy controls [29]. Similarly, Eftekharian et al. identified significant differences in the expression of several miRNAs (miR-211-5p, miR-34a-5p, and miR-204-5p) between healthy controls and responders and non-responders [27].

In contrast, Gonzalez-Martinez et al. employed a prospective longitudinal design with discovery and validation cohorts [13]. They followed patients for two years and differentiated responders and non-responders based on NEDA-3 status. The isolated miRNAs from serum at a single time point between 6 and 12 months after treatment initiation, comparing expression between NEDA-3 and EDA-3 groups at 2 years. They found significantly higher miR-548a-3p expression in the NEDA-3 group across both the discovery and validation cohorts, which demonstrated good predictive performance for identifying patients who achieve NEDA-3 status.

Altogether, miR-548a-3p was the only miRNA directly associated with the response to fingolimod and was validated in an independent cohort. The other significant miRNAs (miR-381-3p, miR-655-3p, miR-211-5p, miR-34a-5p, and miR-204-5p) were reported mainly in comparisons with healthy controls and therefore provide less direct evidence for distinguishing fingolimod responders from non-responders.

#### 3.3.4. Other DMTs

Studies investigating miRNA expression in patients receiving other DMTs are summarized in Table 10. Only one study provided evidence on DMF for the primary outcome [21]. In addition to the primary analysis, miRNA expression was also longitudinally assessed at specific time points. Notably, miR-223-3p and miR-23a-3p were downregulated at 3 and 6 months after treatment initiation, and their baseline levels, as well as their fold changes, could discriminate good from poor responders. Although mir-23b-3p, mir-27a-3p, and miR-27b-3p did not demonstrate a persistent overall change over time, their baseline levels and fold-changes could differentiate responders and non-responders.

Only one study investigated multiple DMTs (natalizumab, IFN, teriflunomide, DMF, ocrelizumab, siponimod, and cladribine) [26]. The key finding was the performance of combined models integrating miRNA expression with extracellular vesicle markers, which showed good discrimination for NEDA-3, NEDA-4, and NEDA-5 statuses. However, because different DMTs were analyzed together, these findings are more informative for classifying treatment outcomes in a treated MS cohort than for identifying biomarkers of response to a specific DMT.

Casanova et al. employed a prospective, longitudinal design with a 5-year follow-up and assessed associations between miRNA expression at baseline and various clinical and radiological outcomes. They identified miR-126-3p and miR-146a-5p as significantly associated with CDP at 2 years, and miR-9-5p and miR-138-5p as significantly associated with EDSS progression and NEDA-3 status, respectively.

### 3.4. Secondary Outcome: Longitudinal Changes in miRNA Expression After Treatment Initiation in Relation to Treatment Response

#### 3.4.1. Overview of the Findings

Four included studies (26.7%) evaluated longitudinal changes in miRNA expression in relation to treatment response [23,24,25,28]. Most followed patients prospectively, with repeated sampling over time, whereas one had a case–control design with more limited temporal assessment [28].

The findings from these studies can be grouped into three main categories: (1) miRNAs that changed their expression mainly in the responder group: miR-26a-5p, miR-130b-3p, miR-654-5p, miR-487b-3p, miR-150-5p, miR-548e-3p; (2) miRNAs that showed modulation shortly after treatment initiation, but without a direct comparison between responders and non-responders: miR-494 and miR-100-5p; and (3) miRNAs whose levels were associated with later disability progression: miR-125a-5p and miR-146a-5p. These studies suggest that the expression of certain miRNAs can be influenced by DMT initiation and, in some cases, these changes are associated with response status or later disability outcomes. However, they do not provide strong evidence on whether these miRNAs can predict treatment response before it becomes clinically evident.

#### 3.4.2. IFN-Based Therapies

De Felice et al. and Magner et al. evaluated longitudinal changes in miRNA expression in patients treated with IFN-β using different approaches [23,28]. Details on these studies are provided in Table 11. De Felice et al. included treatment-naïve patients and analyzed miRNA expression at baseline and at two additional time points (3 and 6 months after treatment initiation). In contrast, Magner et al. evaluated previously treated patients and isolated miRNAs before a scheduled dose of IFN-β and then 48 h after.

De Felice et al. identified only miR-26a-5p as significantly modulated, which was upregulated at 3 months in responders but not non-responders. In contrast, Magner et al. identified several miRNAs with significant changes at 48 h after IFN-β administration, of which miR-494 and miR-100-5p showed higher expression in “good responders” compared to untreated patients.

Altogether, the secondary outcome evidence for IFN-β mainly supports longitudinal changes in miR-26a-5p expression in responders and early changes in miR-494 and miR-100-5p after IFN-β initiation, without direct evidence that these changes were associated with treatment response status.

#### 3.4.3. Other DMTs

Two studies provided evidence on fingolimod and DMF [24,25], and details on them are provided in Table 11. Both used longitudinal designs to assess miRNA dynamics following treatment initiation, but with different outcome indicators.

In patients treated with fingolimod, Ebrahimkhani et al. reported longitudinal changes at 6 months in those with a favorable MRI-based response: miR-130b-3p, miR-654-5p, and miR-487b-3p increased, whereas miR-150-5p and miR-548e-3p decreased [24]. In patients treated with DMF, Giuliani et al. reported that miR-125a-5p and miR-146a-5p were downregulated at 4 months after treatment initiation and were associated with disability progression [25].

### 3.5. Recurrently Reported miRNAs and Findings from ROC Analysis

A few miRNAs have been reported in multiple studies. Among these, miR-23a-3p was associated with treatment response in three studies. Specifically, Carbone et al. found that miR-12a-3p expression was downregulated at 3 and 6 months after treatment initiation (DMF), with baseline levels and fold-changes able to discriminate between “good responders” and “poor responders” [21]. Additionally, Manna et al. observed that miR-23a-3p expression was downregulated in patients treated with IFN-β compared to treatment-naïve patients, observing differential expression across naïve, responder, and non-responder groups [31]. Moreover, Torres-Iglesias et al. found an association between miR-23a-3p expression and NEDA-5 status [26].

In addition, miR-26a-5p was the only miRNA evaluated in two studies investigating the same DMT. De Felice et al. found that miR-26a-5p was upregulated at 3 months after IFN-β initiation in responders, although they did not directly compare responders and non-responders [23]. Additionally, Manna et al. reported that miR-26a-5p was downregulated in patients treated with IFN-β compared to treatment-naïve patients, with changes observed in responders, but did not directly compare responders and non-responders [31].

In contrast, the findings for miR-155 are inconsistent. De Felice et al. found that miR-155 did not change significantly after IFN-β initiation [23], while Giuliani et al. reported that miR-155 expression decreased after treatment with DMF [25]. Similarly, the findings for miR-34a-5p also vary across studies. Eftekharian et al. reported reduced miR-23a-5p expression in responders to fingolimod compared to healthy controls [27], whereas Giuliani et al. found no significant longitudinal changes in miR-23a-5p expression or associations with response to DMF [25].

In addition, miR-146a-5p has been investigated in three studies, in which its expression decreased after DMF treatment and was lower in patients treated with IFN-β than in treatment-naïve patients [25,31]. Giuliani et al. observed that both miR-146a-5p levels at baseline and at 4 months after DMF initiation were associated with disability progression, while Manna et al. observed a modulation in responders. Moreover, Casanova et al. found a significant association between miR-146a-5p expression and CDP at 2 years, but did not assess longitudinal changes in expression [22].

Carbone et al. observed decreased miR-223-3p expression at 3 and 6 months after DMF initiation, with both baseline and fold changes in expression able to discriminate “good responders” from “poor responders” [21]. Moreover, they observed a correlation between miR-223-3p expression and EDSS. Similarly, Manna et al. reported downregulation of miR-223-3p after IFN-β in treated patients compared to treatment-naïve patients, with differential expression across responder groups [31]. However, Casanova et al. found no significant association between miR-223-3p expression and treatment response or clinical outcomes [22].

In a cohort receiving IFN-β1a, Manna et al. showed that miR-660-5p expression increased, with changes seen in responders [31]. However, they did not directly compare responders and non-responders. In addition, Torres-Iglesias et al. found lower miR-660-5p expression in patients with more severe disease activity, as reflected by EDA-4 status [26].

The findings for miR-326 are mixed. Fattahi et al. (2020) found decreased miR-326 expression in IFN-β responders compared with non-responders, although this difference was not significant [30]. Conversely, Torres-Iglesias et al. found reduced miR-326 expression in patients who achieved NEDA-3, NEDA-4, or NEDA-5 status [26].

Among the IFN-β studies that performed receiver operating characteristic (ROC) analysis, Tahmasebivand et al. and Mousavi et al. observed that miR-185-5p and miR-504 only demonstrated a modest ability to distinguish responders from non-responders (area under the ROC curve [AUC] = 0.663 and 0.629, respectively) [32,33]. Gonzalez-Martinez et al. observed better outcomes in patients treated with fingolimod, where miR-548a-3p showed good discrimination between NEDA-3 and EDA-3 groups in the validation cohort (AUC = 0.882) [13]. Torres-Iglesias et al. reported the best performance when combining extracellular vesicles with miRNAs (miR-98-5p, miR-144-5p, and miR-126-5p), where the models reached very high AUCs (0.86 for NEDA-3, 1.00 for NEDA-4, and 0.93 for NEDA-5) [26]. In contrast, although Eftekharian et al. examined several miRNAs, none showed a significant ability to differentiate responders from non-responders [27].

Altogether, although these findings identify miRNAs that warrant further validation, they do not yet confirm them as treatment-response biomarkers. Moreover, while miR-23a-3p, miR-26a-5p, miR-146a-5p, miR-155, miR-34a-5p, miR-223-3p, miR-660-5p, and miR-326 were reported in multiple studies, most were investigated in the context of different DMTs, outcome definitions, or comparison groups. From a practical perspective, the most clinically relevant findings came from studies that associated miRNA expression with clearly defined outcomes and assessed discriminative performance.

### 3.6. Risk of Bias of the Included Studies

The risk of bias in the included studies is summarized in Table 12. Overall, most (13/15, 86.7%) were rated as having a moderate risk of bias, while two (13.3%) were rated as having a low risk of bias. Those assessed using the cohort and case–control versions of the NOS generally scored in the selection and outcome domains, but with insufficient adjustment for confounders and limited group comparability, which remained the most consistent source of potential bias. Among those assessed with the JBI 2017 Critical Appraisal checklist, inclusion criteria, participant description, statistical analysis, exposure measurement, and outcomes were adequately reported, although similar issues with confounding control persisted.

## 4. Discussion

### 4.1. Principal Findings

The available data support the potential role of miRNAs in assessing response to specific DMTs in patients with MS, but it remains insufficient for clinical implementation. The most relevant findings were those supported not only by differences in miRNA expression between response groups but also by clearly defined outcomes, validation, or discriminative performance analysis. Among the 15 included studies, miR-548a-3p in patients treated with fingolimod and miR-223-3p, miR-23a/b-3p, and miR-27a/b-3p in patients treated with DMF are the most relevant targets for future validation, given the more robust methodologies of the studies that reported them. In addition, although miR-29b-3p, miR-29-5p, miR-185-5p, miR-504, and miR-26a-5p showed significant modulation in IFN-β studies, the available evidence was generally more preliminary, with isolated findings and modest discriminative performance in ROC analyses, when performed.

From a clinical perspective, the main significance of our findings is the possibility that miRNAs could help identify patients who are more or less likely to respond to a given DMT. Earlier recognition of suboptimal response may help guide treatment adjustment before ongoing inflammatory activity or disability progression becomes clinically evident. Biologically, most of the miRNAs discussed further in this systematic review are involved in immune and inflammatory regulation, which is central to MS. However, it remains to be established whether the DMT alters miRNA expression directly, whether the change simply follows the reduction in inflammatory activity, or whether the miRNAs themselves help drive the anti-inflammatory effect.

Although numerous studies have investigated miRNA expression in MS, only a few have specifically evaluated their association with treatment response, highlighting an important gap in the current literature.

### 4.2. Heterogeneity of the Included Studies

The most important factor limiting the consistency of results across studies was the high heterogeneity in various methodological aspects. The 15 included studies used various designs, ranging from longitudinal to cross-sectional and case–control, and included both treatment-naïve and previously treated populations.

Another major aspect of heterogeneity was the definition of treatment response, which varied considerably across studies, including the use of clinical parameters (e.g., EDSS progression or number of relapses, either alone or in combination), lesion activity in MRI, or composite outcomes (e.g., NEDA-3), which limits the comparability of results. Although there is currently no single, unanimously accepted definition of treatment response, partly due to clinical and temporal variability in patient characteristics, composite measures such as NEDA have been proposed for use in clinical practice [34,35]. However, several challenges remain in implementing NEDA in clinical settings [36].

Another source of heterogeneity across studies was the different samples from which miRNAs were isolated, ranging from body fluids (plasma, serum, and peripheral whole blood) to PBMCs or exosomes. Notably, each sample type has distinct biological characteristics that may influence miRNA profiles. Exosomes serve as a means of long-distance communication between cells. Secreted by various cell types, they carry and protect miRNAs from ribonuclease degradation, thereby increasing their stability and providing information about their cells of origin [31,37]. In contrast, while cell-free miRNAs from plasma or serum may be easier to quantify, they can be influenced by sample preparation procedures, including miRNAs released from platelets in plasma and from erythrocytes in hemolyzed serum samples [38]. Considering the complex role of miRNAs in regulating activation, differentiation, and proliferation of PBMCs, isolating them from these cells may offer valuable insights into the pathogenesis of MS [9].

In addition, the included studies used different miRNA profiling platforms and normalization strategies, contributing to the heterogeneity across studies. They used different endogenous controls, spike-in methods, or global normalization approaches. RT-qPCR offers high sensitivity for targeted analysis of selected miRNAs. In contrast, broader approaches such as microarrays or RNA sequencing are better suited to discovery, but may introduce greater technical variability and usually require further validation [39].

Finally, differences in sampling time points and follow-up duration further contributed to heterogeneity across studies. In some, miRNA expression was measured only after the established treatment response and compared between responders and non-responders. In these cases, it is difficult to determine whether the observed differences are due to treatment-induced changes in miRNA expression or to underlying disease mechanisms, as miRNAs have previously been suggested to be involved in various pathogenic processes [40]. In contrast, other studies assessed miRNA expression at baseline or included both baseline and follow-up measurements after treatment initiation, thereby enabling better evaluation of their potential association with treatment response.

Overall, our findings are consistent with other reviews that have also highlighted the heterogeneity among studies assessing miRNA expression in MS [16,41].

### 4.3. Methodological Limitations of the Included Studies

One of the most common limitations across the 15 included studies is their relatively small cohorts, which limit the statistical power and generalizability, and increase the risk of false-positive findings. Moreover, other confounding factors are more difficult to adjust for in small cohorts. Specific study designs and small sample sizes may have restricted the use of multivariable analysis. Consequently, many studies relied on unadjusted comparisons. The effects of certain diets and exercises on miRNA expression have been previously studied [42,43]. Other factors, such as aging or smoking, also appear to interfere with the expression of certain miRNAs [44,45].

In addition, many studies employed an exploratory approach. In this context, multiple testing correction was inconsistently applied in studies that simultaneously assessed a large number of miRNAs, thereby increasing the risk of false-positive findings. While some studies specifically acknowledged this limitation, stating the hypothesis-generating nature, others did not clearly report this aspect, which may contribute to overinterpretation of the findings.

The lack of independent validation represents another major limitation across studies. Most studies used a single cohort for analysis and did not include a separate validation cohort; only one study included both a discovery and a validation cohort [13]. This omission limits the robustness and reproducibility of the reported miRNA signatures and their clinical implications as biomarkers of treatment response.

Other isolated limitations, acknowledged by the studies in which they occurred, include treatment changes during the follow-up period and missing samples during follow-up, which may influence the association of miRNAs with treatment response, as well as analyses performed on total extracellular vesicles rather than those specific to a particular origin.

### 4.4. Recurrently Reported miRNAs and Their Biological Relevance

In this section, findings from studies included in this review are discussed in the context of additional evidence from the literature.

A potential protective role for miR-23a has been observed in experimental models, in which it promotes myelination through the differentiation of oligodendrocytes and modulation of certain proteins involved in neuroprotection [46,47]. Supporting this mechanism, miR-23a-3p released from exosomes derived from human umbilical cord mesenchymal stem cells was found to promote oligodendrocyte differentiation and remyelination [48]. However, miR-23a was also found to be overexpressed in monocytes from patients with RRMS [49], consistent with its previously investigated role in macrophage polarization. In addition, Fenoglio et al. observed lower levels of miR-23a in patients with MS compared with healthy controls at baseline, followed by upregulation after initiation of fingolimod [50]. In our systematic review, three included studies suggested that miR-23a-3p may serve as a biomarker for treatment response [21,26,31]. Despite variable results across the investigated DMTs, the repeated changes in miR-23a expression, along with experimental data suggesting its neuroprotective effects, highlight its potential value as a biomarker of treatment response.

miR-155 and its pro-inflammatory role in the pathogenesis of MS have been extensively researched. It was observed to influence the differentiation of Th1 and Th17 cells in an experimental autoimmune encephalomyelitis mouse model, with mice overexpressing miR-155 developing more severe disease [51]. In addition, miR-155 plays a significant role in the activation and modulation of B-cell function and glial cells, promoting BBB disruption and demyelination [52]. Moreover, miR-155 expression was found to be higher in active MS lesions [53]. Furthermore, treating astrocytes with DMF significantly reduced the secretion of pro-inflammatory cytokines, the production of reactive oxygen species, and the expression of miR-155 [54]. In addition, among patients with RRMS, miR-155 expression in monocytes was lower in those treated with DMF than in those not treated, with similar changes observed in patients receiving FTY70 or natalizumab [55]. Moreover, miR-155 expression was found to be higher in patients with MS than in healthy controls, but decreased during and after natalizumab therapy [56]. Notably, interleukin 17A (IL17A) levels were significantly higher in patients showing upregulation of both miR-155 and miR-132. Among the included studies, only Giuliani et al. reported decreased miR-155 expression after initiation of DMF, but did not observe a significant association with disability progression [25]. Although there is substantial evidence supporting the pro-inflammatory role of miR-155, as well as experimental data showing that certain DMTs may reduce miR-155 expression and related pro-inflammatory mechanisms, clinical data evaluating this miRNA in relation to treatment response remain limited.

Along with miR-155 and miR-326, miR-34a is upregulated in active MS lesions and reduces CD47 molecule (CD47) expression, which removes the inhibitory control of macrophages and allows myelin destruction [57]. Conversely, overexpression of miR-34a-5p in CD4^+^ cells downregulated C-X-C motif chemokine receptor 3 (CXCR3), inhibiting Th1 cell polarization and subsequently limiting the activation of cytotoxic T lymphocytes [58]. Supporting its pro-inflammatory role, miR-34a-5p was upregulated in the relapsing phase of patients with MS and could discriminate between patients in the relapsing and remitting phases [59]. Thus, miR-34a-5p may play an inhibitory role in the formation of regulatory T cells and a shift toward Th17 cell differentiation [59]. In addition, a prospective clinical study observed associations between miR-34a-5p and active brain and spinal cord lesions in MRI, as well as correlations with reduced brain volume [60]. Moreover, Agostini et al. suggested the potential of miR-34a-5p to discriminate patients with RRMS who are more likely to convert to SPMS, observing increased serum levels of miR-34a-5p several years before conversion [18]. Furthermore, Eftekharian et al. found that responders to fingolimod had lower levels of miR-34a-5p than healthy controls [27]. Although evidence regarding treatment response remains limited, available findings suggest that miR-34a-5p is more closely associated with inflammatory disease activity than with a clearly defined response to a specific DMT.

Although the role of miR-660 in the pathogenesis of other diseases has been explored, it is less well characterized in the context of MS. Increased miR-660-5p levels have been observed in responders to IFN-β1a, although responder groups were not directly compared [31]. In addition, lower miR-660-5p levels have been associated with greater disease activity [26]. Moreover, miR-660-5p levels were lower in untreated patients with MS than in healthy controls and were significantly upregulated after treatment with DMF [61]. Overall, the available data from clinical studies suggest that miR-660-5p expression is lower in more active or untreated MS but is higher after DMT exposure. Thus, miR-660-5p warrants further research on treatment-related changes, although the current evidence is insufficient for treatment-response classification.

The role of miR-326 in the pathogenesis of MS and its clinical relevance as a diagnostic and discriminatory biomarker have been previously studied. In an experimental mouse model, miR-326 was associated with MS severity, targeting ETS proto-oncogene 1 transcription factor (*Ets1*) and thereby promoting Th-17 differentiation [62]. In addition, miR-326 is upregulated in active MS lesions and promotes macrophage-mediated myelin destruction by reducing CD47 expression on brain cells [57]. Consistent with these findings, miR-326 expression was significantly higher in PBMCs, PBLs, and T-cell-derived exosomes from patients with RRMS compared with healthy controls [14,62,63]. Al-Temaimi et al. suggested that miR-326 could serve as a diagnostic biomarker for patients with RRMS, as its expression was lower in patients with RRMS than in healthy controls and was associated with lower brain-derived neurotrophic factor (BDNF) levels [64]. In addition, miR-326 was upregulated in the relapsing phase of patients with MS and could significantly discriminate between patients in the relapsing and remitting phases [65]. Moreover, miR-326 was upregulated by cladribine treatment [66] but downregulated by natalizumab treatment [12]. Regarding treatment response, two included studies investigated miR-326. Fattahi et al. observed nonsignificantly lower miR-326 expression in responders to IFN-β, while Torres-Iglesias et al. observed lower miR-326 expression in patients who achieved NEDA-3, NEDA-4, or NEDA-5 [26,30]. Overall, miR-326 appears more closely associated with inflammatory activity and disease severity in MS than with treatment response, which may explain why lower miR-326 levels were reported in patients with more favorable outcomes across included studies. However, because different DMTs have been associated with different directions of change, further research is needed before miR-326 can be interpreted as a biomarker of treatment response.

The possible involvement of miR-26a-5p in the pathogenesis of MS has been investigated through experimental and in silico analyses. The findings suggest roles in regulating glutamate transport via solute carrier family 1 member 1 (SLC1A1) and in Th17 cell differentiation through the transforming growth factor–beta (TGF-β) signaling pathway [65,67]. Mameli et al. observed that miR-26a expression was downregulated after 6 months of natalizumab therapy, with higher miR-26a expression associated with increased levels of pro-inflammatory cytokines, including interferon gamma (IFNG/IFN-γ) and tumor necrosis factor (TNF/TNF-α) [56]. Moreover, miR-26a-5p expression was increased after cladribine treatment [68]. In contrast, the included studies focused specifically on miR-26a-5p in the context of IFN-β treatment and reported inconsistent results [23,31]. Considering these findings, miR-26a-5p highlights that post-treatment changes in miRNA expression do not automatically translate into a response biomarker. Although its expression appears to be influenced by several DMTs, it cannot be concluded whether these changes reflect a direct clinical effect of treatment or broader changes in inflammatory activity.

miR-146a is a widely studied miRNA with both pro- and anti-inflammatory roles in the pathogenesis of MS. Its anti-inflammatory effects are mainly associated with the regulation of the nuclear factor kappa B (NF-κB) signaling pathway, including inhibition of leukocyte adhesion and promotion of microglial polarization toward the alternatively activated (M2) phenotype [69,70,71]. Another proposed mechanism by which miR-146a may limit inflammation is by reducing interleukin 6 (IL6) and 21 (IL21) production, thereby decreasing Th17 differentiation [72]. Moreover, miR-146a may have a therapeutic effect in MS by reducing demyelination and promoting remyelination [73,74]. In contrast, miR-146a may have a pro-inflammatory role, as mice deficient in miR-146a showed reduced inflammatory responses and demyelination [75]. These experimental findings highlight the complex and context-dependent role of miR-146a and may help explain the variability observed in clinical studies. CSF levels of miR-146a were increased in patients with active MS lesions, suggesting a role as a biomarker of inflammatory activity [76]. Similarly, miR-146a levels were higher in untreated patients than in healthy controls [14], but lower in patients with MS than in controls [56]. Increased expression of miR-146a-5p and other NF-κB-related miRNAs has been observed following treatment with DMF [61]. In addition, miR-146a-5p levels have been associated with clinical outcomes (EDSS and Symbol Digit Modalities Test) [77]. Overall, both experimental and clinical studies provide valuable evidence supporting miR-146a as a relevant biomarker of MS progression and disability, showing post-treatment changes, as also reflected in the studies included in this review [22,25,31], although the direction of these changes remains unclear.

In experimental models, miR-223 has been shown to modulate immune responses and may exert both anti-inflammatory and pro-inflammatory effects. It induces macrophage polarization toward an M2 phenotype by targeting signal transducer and activator of transcription 3 (STAT3) [78]. Moreover, it appears to be upregulated in response to inflammation and modulates glutamate signaling pathways [79]. However, Satoorian et al. suggested that miR-223 may promote Th1 and Th17 differentiation [80]. Clinical studies further support the role of miR-223 as a dynamic marker of immune activity. Orefice et al. observed changes in miR-223-3p expression after 6 months of ocrelizumab treatment along with a negative correlation between miR-223-3p and IL-6 [81]. Moreover, miR-223-3p levels showed longitudinal changes over 4 years, with its temporal variability associated with relapse activity [82]. Additionally, Dominguez-Mozo et al. identified a positive correlation between miR-223-3p and caudate volume [77]. Moreover, in patients treated with fingolimod, miR-223 levels were lower at baseline compared to healthy controls and increased toward normal levels after treatment [50]. In the case of miR-223-3p, experimental and clinical data converge on a role in immune regulation and treatment response, with repeated changes in expression following fingolimod, IFN-β [31], and DMF [21], although the direction of these changes varies across studies and treatments. A distinctive feature of miR-223-3p is its association with relapse activity and its ability to discriminate between responder groups [21], suggesting a potential role as a biomarker of treatment response that warrants further validation.

Although the studies included in this systematic review examined various miRNAs, modulated in different directions, common targets and pathways were observed. One example of a shared molecular target is CD47: in a single profiling study of MS lesions, miR-326, miR-34a, and miR-155 were found to reduce CD47 expression, thereby removing the inhibitory control of macrophages and facilitating myelin destruction [57]. Although derived from a single study, these findings illustrate how distinct miRNAs may act on a shared target relevant to demyelination. Regarding immune mechanisms, several miRNAs share common pathways through different targets. Th17 cell differentiation is a clear example, being influenced by miR-326 via *Ets1* [62], by miR-26a-5p via the TGF-β signaling pathway [67], and by miR-146a via IL6 and IL21 [72]. Another example is the polarization of macrophages and microglia toward the M2 phenotype, regulated by miR-223 via STAT3 [78] and by miR-146a [70]. The NF-κB signaling pathway serves as a key regulator of immune responses in MS: miR-146a and miR-155 have been described as NF-κB-sensitive miRNAs, which may reflect a shared molecular mechanism across patients with MS [83]. Overall, this finding suggests that the mixed results observed in the direction of miRNA modulation may, at least in part, be accompanied by shared underlying inflammatory and demyelinating pathways central to MS pathology, supporting the value of evaluating functionally related miRNA panels, rather than single molecules, in future studies.

### 4.5. Implications for Future Research and Potential Clinical Applications

Although there is increasing evidence on the role of miRNAs as promising biomarkers of treatment response in MS, several methodological and biological challenges remain when interpreting the results across studies. Firstly, robust prospective study designs with larger populations, including both discovery and validation cohorts, should be considered to identify and validate clinically applicable predictive and monitoring biomarkers. This shift would enable proper statistical multivariable analysis with appropriate adjustment for confounding factors. Whether the studies are confirmatory or exploratory, appropriate adjustments should be applied for multiple testing, balancing the risks of false-positive and false-negative discoveries [84].

Secondly, baseline sampling followed by longitudinal sampling after DMT initiation in treatment-naïve patients, along with valid extraction methods and appropriate normalization strategies, is recommended to better capture dynamic changes in miRNA levels in response to treatment. Differentiating responders from non-responders based on composite clinical and radiological measures, such as NEDA, would allow for a more standardized assessment of treatment response and improve identification of miRNA signatures associated with therapeutic outcomes.

Thirdly, further research on miRNAs isolated from exosomes derived from specific cell types would not only enhance understanding of the biological roles of specific miRNAs but also provide valuable information regarding disease mechanisms and molecular changes induced by certain DMTs. It is important to confirm whether changes in miRNA expression are due to treatment or reflect secondary effects of the disease itself. Moreover, future studies should focus on miRNA expression in patients receiving high-efficacy therapies, as these treatments may induce more pronounced and targeted molecular changes in the context of more active disease.

Fourthly, in the context of personalized treatment, both disease progression and treatment response should be predicted. Along with other molecular and imaging biomarkers, several miRNAs have been proposed to help predict MS progression, which could help identify patients with RRMS at risk of transitioning to SPMS and thereby support earlier treatment decisions [64,85]. In addition, assessing predefined miRNA panels as potential predictive biomarkers of treatment response would provide an important direction with potential clinical implications in personalized treatment. Instead of relying solely on broad exploratory profiling, these panels should be selected based on recurrent findings and biological relevance, as well as on previous research reporting predictive performances. They should be tested in prospective studies with baseline and follow-up sampling and confirmed in independent validation cohorts.

### 4.6. Limitations of This Systematic Review

This systematic review had some limitations that should be acknowledged when interpreting the findings. Firstly, the small number of studies investigating the same DMT and the marked heterogeneity in study designs, patient populations, definition of treatment response, and analytical methods limited the comparability of results and the ability to perform a meta-analysis. Secondly, most included studies evaluated miRNAs in relation to platform therapies, which may have been administered to patients with earlier or less active MS stages, compared to those treated with higher-efficacy therapies, which may limit the identification of consistent and reproducible biomarkers across studies. Thirdly, there may be overlap between study populations, and many studies were conducted in a limited geographic setting, which may affect the generalizability of the findings. Fifthly, this systematic review represents an early synthesis of a still limited body of evidence. The small number of available studies, as well as the fact that for several DMTs the evidence was based on single studies, limits the strength of treatment-specific conclusions. Finally, the included studies measured miRNAs from different biological sources, including serum or plasma, PBMCs or PBLs, whole blood, and extracellular vesicles. Because miRNA expression may vary across biological compartments, the findings from one sample type cannot be directly extrapolated to another.

## 5. Conclusions

The existing literature on associations between miRNA expression and treatment response provides valuable but limited and heterogeneous evidence of their promising role as biomarkers of therapeutic response in patients with MS. Heterogeneity in study designs, definitions of treatment response, and miRNA isolation and analysis methods prevents the identification of clinically applicable and validated miRNAs. Despite these limitations, miR-548a-3p in fingolimod-treated patients and miR-223-3p, miR-23a/b-3p, and miR-27a/b-3p in DMF-treated patients are the most relevant targets for future validation. In contrast, IFN-β-related miRNAs, including miR-29b-3p, miR-29-5p, miR-185-5p, miR-504, and miR-26a-5p, remain more preliminary. When considering studies within the same DMT context, miR-26a-5p was the only miRNA assessed in more than one IFN-β-related analysis; however, the current data are insufficient to support it as a reproducible biomarker of IFN-β response. Overall, miRNAs should currently be considered exploratory biomarkers, and further studies with independent validation are required before they are translated into clinical applications.

## Figures and Tables

**Table 1:** Characteristics of the included prospective longitudinal observational studies.

Author	Country	Follow-Up Duration	Analysis Approach
Carbone et al. 2025 [21]	Italy	2 years	Baseline miRNA levels compared between “good” and “poor” DMF responders defined by EDSS progression; longitudinal miRNA changes assessed relative to baseline (3–24 months); treatment-induced fold changes were compared between responder groups
Casanova et al. 2023 [22]	Spain	5 years	Association between baseline miRNA levels and 2- and 5-year clinical and radiological outcomes (CDP, NEDA-3, MRI activity)
De Felice et al. 2014 [23]	Italy	6 months	Longitudinal change in miRNA expression after IFN-β initiation, analyzed separately in responders and non-responders.
Ebrahimkhani et al. 2020 [24]	Australia	1 year	Longitudinal change in miRNA expression after FTY720 initiation, analyzed within “responders” group (baseline vs. 6-month follow-up)
Giuliani et al. 2021 [25]	Italy	1 year	Longitudinal change in miRNA expression after DMF initiation (baseline vs. 4-month follow-up), with subgroup analysis according to disability progression
Gonzalez-Martinez et al. 2023 [13]	USA	2 years	miRNA expression in FTY720 treated patients with NEDA-3 vs. EDA-3 at 2 years
Torres-Iglesias et al. 2025 [26]	Spain	1 year	miRNA expression compared between responders (NEDA-3/NEDA-4/NEDA-5) and non-responders (EDA-3/EDA-4/EDA-5)

CDP, confirmed disability progression; DMF, dimethyl fumarate; EDA-3, evidence of disease activity–3 status; EDSS, Expanded Disability Status Scale; FTY720, fingolimod; IFN, interferon; miRNA, microRNA; MRI, magnetic resonance imaging; NEDA-3, no evidence of disease activity–3 status.

**Table 2:** Characteristics of the included observational case–control studies.

Author	Country	Follow-Up Duration	Analysis Approach
Eftekharian et al. 2019 [27]	Iran	2 years	miRNA expression compared between HC and subgroups of MS patients and between FTY720 responders and non-responders
Magner et al. 2019 [28]	USA	48 h	miRNA expression compared between treated (“good”/“poor” responders) and untreated MS patients and pre-IFNβ1a vs. 48 h post-injection
Mazdeh et al. 2020 [29]	Iran	≥6 months prior to sampling (used to define treatment response)	miRNA expression compared between FTY720 responders, non-responders and HC

FTY720, fingolimod; HC, healthy control; IFN, interferon; miRNA, microRNA; MS, multiple sclerosis.

**Table 3:** Characteristics of the included cross-sectional studies.

Author	Country	Follow-Up Duration	Analysis Approach
Fattahi et al. 2019 [15]	Iran	≥1 year prior to sampling (used to define treatment response)	miRNA expression compared between IFN-β responders and non-responders and between HC and subgroups of MS patients
Fattahi et al. 2020 [30]	Iran	≥1 year prior to sampling (used to define treatment response)	miRNA expression compared between IFN-β responders and non-responders and between HC and subgroups of MS patients
Manna et al. 2018 [31]	Italy	≥2 years prior to sampling (used to define treatment response)	miRNA expression compared across treatment-naïve, IFN-β–treated responders, and non-responders
Mousavi et al. 2020 [32]	Iran	≥1 year prior to sampling (used to define treatment response)	miRNA expression compared between IFN-β1a responders and non-responders
Tahmasebivand et al. 2020 [33]	Iran	≥1 year prior to sampling (used to define treatment response)	miRNA expression compared between IFN-β responders and non-responders

HC, healthy control; IFN, interferon; miRNA, microRNA; MS, multiple sclerosis.

**Table 4:** Patients’ characteristics.

Author	Number of Patients	Patients at Each Timepoint	Age ^¥^	F/M	Disease Duration °	Baseline EDSS ^■^
Carbone et al. 2025 [21]	19 DMF 19 HC	T0 = 19 T1(3 m) = 11 T2(6 m) = 15 T3(12 m) = 10 T4(24 m) = 13	34.6 ± 2.6	13:06	N/R	2.0 ± 0.84
	8 R, 11 NR					
Casanova et al. 2023 [22]	26 GA	T0 = 26 T1(2 y) = 25 T2(5 y) = 24	32.8 [26.6–44.9]	18:08	N/R	1.4 ± 1.7
De Felice et al. 2014 [23]	50 IFN-β	T0 = 50 T1(3 m) = 50 T2(6 m) = 50	29 ± 3	18:22	4 ± 2	2.8 ± 2
	40 R, 10 NR					
Ebrahimkhani et al. 2020 [24]	29 FTY720	T0 = 29 T1(6 m) = 29 T2(12 m) = 29	38.6 ± 10.2	17:12	4.9 (0.1–26.5)	1.7 ± 1.3
Eftekharian et al. 2019 [27]	78 FTY720 79 HC	78 (single timepoint)	31.12 ± 8.18	58:20	5.2 ± 4.5	3.25 ± 1.15
	49 R, 29 NR					
Fattahi et al. 2019 [15]	70 IFN-β, 20 HC	70 (single timepoint)	33.72 ± 8.19 (R)	30:5 (R)	N/R	0–5 (R)
	35 R, 35 NR		35.44 ± 8.06 (NR)	29:6 (NR)		0–5 (NR)
Fattahi et al. 2020 [30]	70 IFN-β	70 (single timepoint)	33.72 ± 8.19 (R)	30:5 (R)	N/R	0–5 (R)
	35 R, 35 NR		35.44 ± 8.06 (NR)	29:6 (NR)		0–5 (NR)
Giuliani et al. 2021 [25]	24 DMF 21 HC	T0 = 24 T1(4 m) = 16	42 [36–45]	14:10	4 [2–10]	1.5 [1–3]
Gonzalez-Martinez et al. 2023 [13]	53 FTY720	53 (single timepoint)	42.2 ± 12.20	40:13	N/R	1.64 ± 1.15
	31 discovery					
	22 validation					
Magner et al. 2019 [28]	50 IFN-β 24 NTZ 25 HC	T0 = 50 T1 (48 h post-IFNβ1a) = 50	54 ± 7 (R)	21:4 (R)	18 ± 8.8 (R)	2.3 (0.8)
	25 R, 25 NR (IFN-β)		51 ± 11 (NR)	20:5 (NR)	18.1 ± 11.1 (NR)	4.4 (2.1)
Manna et al. 2018 [31]	11 IFN-β	11 (single timepoint)	33.0 ± 3.5 (R)	2:2 (R)	N/R	1.5–2
	4 R, 3 NR		37.3 ± 6.8 (NR)	3:0 (NR)		4–6
Mazdeh et al. 2020 [29]	78 FTY720 79 HC	78 (single timepoint)	36.28 ± 8.8	58:20	5.2 ± 4.5	3.25 ± 1.15
	49 R, 29 NR					
Mousavi et al. 2020 [32]	100 IFN-β	100 (single timepoint)	34.48 ± 8.89 (R)	38:12 (R)	5.24 ± 4.90 (R)	1.5 ± 0.6
	50 R, 50 NR		34.08 ± 9.34 (NR)	41:9 (NR)	6.79 ± 4.56 (NR)	3 ± 2.07
Tahmasebivand et al. 2020 [33]	105 IFN-β	105 (single timepoint)	34.30 ± 8.76 (R)	34:18 (R)	5.03 ± 4.91 (R)	1.5 ± 0.6 (R)
	52 R, 53 NR		34.16 ± 9.5 (NR)	44:9 (NR)	6.59 ± 4.50 (NR)	3 ± 2.07 (NR)
Torres-Iglesias et al. 2025 [26]	80 various DMT	T0 = 80; T1(3 m) = 80; T2(12 m) = 80	44.06 ± 9.03 (R)	25:24 (R)	10.29 ± 10.04 (R)	1.89 ± 2.07 (R)
	49 R, 31 NR		43.82 ± 11.23 (NR)	18:13 (NR)	12.77 ± 11.41 (NR)	2 ± 2.29 (NR)

DMT, disease-modifying therapy; DMF, dimethyl fumarate; F, female; FTY720, fingolimod; GA, glatiramer acetate; HC, healthy control; IFN, interferon; M, male; NR, non-responder; N/R, not reported; NTZ, natalizumab; R, responders. ^¥^ Expressed in years as mean ± standard deviation (SD) or median [interquartile range (IQR)]. ° Expressed in years as mean ± standard deviation (SD) or range, or median [IQR]. ^■^ Expressed as mean ± SD, or median [IQR] or range.

**Table 5:** Treatment exposure and prior treatment characteristics.

Author	Treatment	Treatment-Naïve Patients	Previously Treated at Study Entry	Washout Period
Carbone et al. 2025 [21]	DMF	19	0	N/A
Casanova et al. 2023 [22]	GA	0	26 GA	N/A
De Felice et al. 2014 [23]	IFN β	50	0	N/A
Ebrahimkhani et al. 2020 [24]	FTY720	29	0	N/A
Eftekharian et al. 2019 [27]	FTY720	N/R	N/R	N/R
Fattahi et al. 2019 [15]	IFN β	0	70 IFN β	N/A
Fattahi et al. 2020 [30]	IFN β	0	70 IFN β	N/A
Giuliani et al. 2021 [25]	DMF	8	16	2 weeks IFN-β/GA 4 weeks FTY720/NTZ
Gonzalez-Martinez et al. 2023 [13]	FTY720	6	47	N/R
Magner et al. 2019 [28]	IFN β1a	0	50	N/A
	NTZ			
Manna et al. 2018 [31]	IFN β1a	4	7	N/R
Mazdeh et al. 2020 [29]	FTY720	0	78	N/A
Mousavi et al. 2020 [32]	IFN β1a	0	100	N/A
Tahmasebivand et al. 2020 [33]	IFN β	0	105	N/A
Torres-Iglesias et al. 2025 [26]	NTZ, Teriflunomide, IFN, DMF, Ocrelizumab, Siponimod, Cladribine	N/R (mixed cohort)	N/R (some patients switched DMT)	According to clinical practice

DMF, dimethyl fumarate; DMT = disease-modifying therapy; FTY720, fingolimod; GA, glatiramer acetate; IFN-β, interferon-β; N/A, not applicable; N/R, not reported; NTZ, natalizumab.

**Table 6:** Definition of treatment response.

Author	Definition of Treatment Response
Carbone et al. 2025 [21]	Decrease in EDSS over the 2 years following treatment initiation
Casanova et al. 2023 [22]	CDP (6-month confirmed EDSS increase), MRI activity (≥1 Gd-enhancing lesion and/or ≥2 new or enlarging T2 lesions), new relapses, NEDA-3
De Felice et al. 2014 [23]	No increase in EDSS score and no relapses during follow-up
Ebrahimkhani et al. 2020 [24]	MRI activity (Gd-enhancing lesions) “Stable responders”: no Gd-enhancing lesions at baseline and 6 months “Positive responders”: active at baseline and quiescent at 6 months
Eftekharian et al. 2019 [27]	No increase in EDSS and no relapse during 2-year follow-up
Fattahi et al. 2019 [15]	No increase in EDSS and no relapse during follow-up
Fattahi et al. 2020 [30]	No increase in EDSS and no relapse during follow-up
Giuliani et al. 2021 [25]	Disability progression defined by EDSS/ARMSS change at 12 months
Gonzalez-Martinez et al. 2023 [13]	NEDA-3 at 2 years follow-up (no EDSS progression, no relapses, no MRI activity)
Magner et al. 2019 [28]	No MRI or clinical activity for at least 2 years on treatment
Manna et al. 2018 [31]	No increase in EDSS, no relapse and no MRI activity during follow-up
Mousavi et al. 2020 [32]	No increase in EDSS, no relapse and no MRI activity during follow-up
Tahmasebivand et al. 2020 [33]	No relapses and no MRI activity during follow-up
Torres-Iglesias et al. 2025 [26]	NEDA-3/NEDA-4/NEDA-5 at 12-month follow-up

ARMSS, age-related multiple sclerosis severity; CDP, confirmed disability progression; EDSS, Expanded Disability Status Scale; Gd, gadolinium; MRI, magnetic resonance imaging; NEDA, no evidence of disease activity.

**Table 7:** Sample types and analytical methods.

Author	Sample Type	Timepoint of Sampling	miRNA Profiling Method	Normalization Strategy	Multiple Testing Correction
Carbone et al. 2025 [21]	Plasma	Baseline + longitudinal (3, 6, 12, 24 m)	qRT-PCR	Global mean normalization	N/R
Casanova et al. 2023 [22]	Serum	Baseline only	Targeted qRT-PCR	Mean of endogenous controls (miR-191-5p, miR-30c-5p)	N/R
De Felice et al. 2014 [23]	PBMCs	Baseline + longitudinal (3, 6 m)	Small RNA cloning and sequencing followed by targeted qRT-PCR validation	Endogenous control RNU6B	N/R
Ebrahimkhani et al. 2020 [24]	Serum Exosomes	Baseline + 6 m	Small RNA sequencing	Limma-based	No (for miRNA differential expression)
Eftekharian et al. 2019 [27]	Peripheral whole blood	Single timepoint (not specified)	qRT-PCR	Endogenous control RNU6B	N/R
Fattahi et al. 2019 [15]	PBMCs	Single timepoint (≥1 year after IFN-β initiation)	qRT-PCR	Endogenous control RUN48 and SNORD44	N/R
Fattahi et al. 2020 [30]	PBMCs	Single timepoint (≥1 year after IFN-β initiation)	qRT-PCR	Endogenous control RUN48	N/R
Giuliani et al. 2021 [25]	Plasma	Baseline + 4 m	qRT-PCR	Spike-in cel-miR-39 normalization	N/R
Gonzalez-Martinez et al. 2023 [13]	Serum	Single timepoint (6–12 m after FTY720 initiation)	LNA SYBR Green-based RT-qPCR miRNA panel (Exiqon) with RT-qPCR validation	NormFinder	N/R
Magner et al. 2019 [28]	PBLs	Longitudinal (prior + 48 h after a scheduled IFN-β dose)	Small RNA sequencing	Not explicitly reported (edgeR used for analysis)	FDR
Manna et al. 2018 [31]	Serum Exosomes	Single timepoint (after ≥2 years of therapy)	qRT-PCR; validation by miRCURY LNA RT-qPCR	Spike-in cel-miR-39 normalization	N/R
Mazdeh et al. 2020 [29]	Peripheral whole blood	Single timepoint (≥6 months after FTY720 initiation)	qRT-PCR	RNU6B normalization	N/R
Mousavi et al. 2020 [32]	Peripheral whole blood	Single timepoint (≥12 months after IFN-β initiation)	qRT-PCR	Endogenous control U6 snRNA	N/R
Tahmasebivand et al. 2020 [33]	Peripheral whole blood	Single timepoint (≥12 months after IFN-β initiation)	qRT-PCR	Endogenous control U6 snRNA	N/R
Torres-Iglesias et al. 2025 [26]	Plasma extracellular vesicles	Baseline + 3 m	Small RNA sequencing	DESeq2 normalization	FDR (implicit in DESeq2)

FDR, false discovery rate; FTY720, fingolimod; IFN, interferon; m, months; N/R = not reported; PBMC, peripheral blood mononuclear cell; PBL, peripheral blood leukocyte; qRT-PCR, reverse transcription–quantitative polymerase chain reaction.

**Table 8:** Studies evaluating miRNAs as biomarkers of the response to IFN-β.

Author	Relevant Investigated miRNA	Change in Expression After Treatment	Statistical Tests	*p* Value	AUC	Association with Treatment Response
Fattahi et al. 2019 [15]	miR-29b-3p	N/A	*t*-test	*p* < 0.05	N/P	↓ in R vs. NR (~3.5-fold)
	miR-29b-5p	N/A		*p* < 0.05		↓ in R vs. NR (~3-fold)
Fattahi et al. 2020 [30]	miR-326	N/A	*t*-test	*p* = 0.7	N/P	No significant association (lower expression reported in R vs. NR)
Manna et al. 2018 [31]	miR-22-3p	↑ in IFN-β–treated vs. treatment-naïve	*t*-test, one-way ANOVA	*p* ^¥^ ≤ 0.001	N/P	↑ after treatment; modulation observed in responders; no direct R vs. NR comparison
	miR-146a-5p	↓ in IFN-β–treated vs. treatment-naïve		*p* ^¥^ ≤ 0.05		↓ after treatment; modulation observed in responders; no direct R vs. NR comparison
	miR-23a-3p	↓ in IFN-β–treated vs. treatment-naïve		*p* ^¥^ ≤ 0.001		↓ after treatment; modulation observed in responders; no direct R vs. NR comparison
	miR-223-3p	↓ in IFN-β–treated vs. treatment-naïve		*p* ^¥^ ≤ 0.001		↓ after treatment; modulation observed in responders; no direct R vs. NR comparison
	miR-26a-5p	↓ in IFN-β–treated vs. treatment-naïve		*p* ^¥^ ≤ 0.05		↓ after treatment; modulation observed in responders; no direct R vs. NR comparison
	miR-660-5p	↑ in IFN-β–treated vs. treatment-naïve		*p* ^¥^ ≤ 0.001		↑ after treatment; modulation observed in responders; no direct R vs. NR comparison
Mousavi et al. 2020 [32]	miR-185-5p	N/A	*t*-test	*p* = 0.001	0.6635 (R vs. NR)	Significantly ↓ in R vs. NR
	miR-320a	N/A		*p* = 0.169		No significant difference in the expression between R and NR
Tahmasebivand et al. [33] 2020	miR-504	N/A	*t*-test	*p* = 0.008	0.629 (R vs. NR)	↑ expression in R vs. NR
	miR-711	N/A		*p* = 0.29		No significant difference in the expression between R and NR

AUC, area under the receiver operating characteristic curve; N/A, not assessed; N/P, not performed; NR, non-responder; R, responder; ↑, upregulated; ↓, downregulated. ^¥^ Reported for one-way analysis of variance (ANOVA) of naïve vs. responders vs. non-responders.

**Table 9:** Studies evaluating miRNAs as biomarkers of response to fingolimod.

Author	Relevant Investigated miRNA	Change in Expression After Treatment	Statistical Tests	*p* Value	AUC	Association with Treatment Response
Eftekharian et al. 2019 [27]	miR-96-5p	N/A	Bayesian multilevel model; ROC analysis	ns		ns difference between HC and R
	miR-211-5p	N/A		*p* = 0.004 (R); 0.018 (NR); 0.017 (F R)	0.543 (R vs. NR)	↓ in both R and NR compared to HC; ↓ in F R compared to F HC
	miR-15a	N/A		ns		ns association
	miR-34a-5p	N/A		*p* = 0.013 (R)	0.556 (R vs. NR)	↓ in R compared to HC
	miR-204-5p	N/A		*p* = 0.04 (M NR)	0.614 (R vs. NR)	↓ in M NR compared to M HC
	miR-501-5p	N/A		ns		ns association
	miR-524-5p	N/A		ns		ns association
Gonzalez-Martinez et al. 2023 [13]	miR-548a-3p	N/A	Wilcoxon rank sum test; proportional odds model; ROC analysis	*p* = 0.04 (discovery); *p* = 0.03 (validation)	0.882 (in validation cohort-NEDA-3 vs. EDA-3)	↑ expression in NEDA-3 vs. EDA-3
Mazdeh et al. 2020 [29]	miR-381-3p	N/A	Bayesian multilevel model	*p* = 0.005	N/P	ns difference in the expression of miRNA between R and NR; ↑ in R vs. HC
	miR-655-3p	N/A		*p* = 0.014, *p* < 0.0001		ns difference in the expression of miRNA between R and NR; ↓ in R and NR vs. HC

AUC, area under the receiver operating characteristic curve; EDA, evidence of disease activity; F, female; HC, healthy control; M, male; N/A, not assessed; N/P, not performed; NEDA, no evidence of disease activity; NR, non-responder; ns, not significant; R, responder; ROC, receiver operating characteristic; ↓, downregulated; ↑, upregulated.

**Table 10:** Studies evaluating miRNAs as biomarkers of response to other DMTs.

Author	miRNA	Change in Expression After Treatment	Statistical Tests	*p* Value	AUC	Association with Treatment Response
Carbone et al. 2025 [21]	miR-223-3p	↓ at 3–6 months	*t*-test/Mann–Whitney; paired comparisons for longitudinal change; correlation analyses	*p* ≤ 0.05 °	N/P	Baseline + fold change discriminate good vs. poor R
	miR-23a-3p	↓ at 3–6 months		*p* ≤ 0.05 °		Baseline + fold change discriminate R
	mir-23b-3p	No persistent overall modulation		*p* ≤ 0.05 °		Baseline + fold change discriminate R
	mir-27a-3p	No persistent overall modulation		*p* ≤ 0.05 °		Baseline + fold change discriminate R
	miR-27b-3p	No persistent overall modulation		*p* ≤ 0.05 °		Baseline + fold change discriminate R
Casanova et al. 2023 [22]	miR-9-5p	N/A	Mann–Whitney U; backstep multivariate regression	*p* = 0.047 ^¥^	N/P	Significant association with EDSS progression at 2 years
	miR-126-3p	N/A		*p*= 0.05 °		Significant association with CDP at 2 years
	miR-138-5p	N/A		*p* = 0.033 °		Significant association with NEDA-3 at 2 years
	miR-146a-5p	N/A		*p* = 0.044 °		Significant association with CDP at 2 years
	miR-200c-3p	N/A		ns °		No significant association
	miR-223-3p	N/A		ns °		No significant association
Torres-Iglesias et al. 2025 [26]	miR-28-3p	N/A	DESeq2 differential expression analysis; logistic regression; ROC analysis	*p* ≤ 0.05		↓ in patients with NEDA-3/NEDA-4/NEDA-5
	miR-326	N/A		*p* ≤ 0.05		↓ in patients with NEDA-3/NEDA-4/NEDA-5
	mir-98-5p	N/A		*p* ≤ 0.05	0.86 (combined model, NEDA-3)	↓ in patients with EDA-3/EDA-4/EDA-5
	miR-144-5p	N/A		*p* ≤ 0.05	1.0 (combined model, NEDA-4)	↓ in patients with EDA-3/EDA-4/EDA-5
	miR-660-5p	N/A		*p* ≤ 0.05		↓ in patients with EDA-4
	miR-222-3p	N/A		*p* ≤ 0.05		↓ in patients with EDA-4
	miR-23a-3p	N/A		*p* ≤ 0.05		associated with NEDA-5
	miR-574	N/A		*p* ≤ 0.05		↓ in patients with EDA-3/EDA-4/EDA-5
	miR-126-5p	N/A		*p* ≤ 0.05	0.93 (combined model, NEDA-5)	↓ in patients with NEDA-3/NEDA-4/NEDA-5
	miR-4433b	N/A		*p* ≤ 0.05		↓ in patients with NEDA-3/NEDA-4/NEDA-5
	miR-660	N/A		*p* ≤ 0.05		↓ in patients with EDA-3/EDA-4/EDA-5

AUC = area under the receiver operating characteristic curve; CDP, confirmed disability progression; EDA, evidence of disease activity; EDSS, Expanded Disability Status Scale; HC, healthy control; N/A, not assessed; N/P, not performed; NEDA, no evidence of disease activity; NR, non-responders; R, responder; ↓, downregulated. ° Reported for a Mann–Whitney *U* test. ^¥^, Reported for backstep multivariate regression.

**Table 11:** Studies evaluating miRNAs as biomarkers of the response to DMTs.

Author	miRNA	Change in Expression After Treatment	Statistical Tests	*p* Value	AUC	Association with Treatment Response
De Felice et al. 2014 [23]	miR-26a-5p	↑ at 3 months in R	One-way ANOVA + Student–Newman–Keuls	*p* < 0.05	N/P	Longitudinal change observed in R, but not in NR; no direct R vs. NR test
	miR-155	ns		ns		
	miR-3676	ns		ns		
	miR-18b	ns		ns		
	miR-599	ns		ns		
	miR-326	ns		ns		
Magner et al. 2019 [28]	miR-145	Expression normalized after IFNβ1a treatment	edgeR differential expression analysis	N/R for R vs. NR	N/P	No association with treatment response (normalized in treated patients regardless of response)
	miR-494	↓ in GR after treatment (restored toward HC levels)		*p* = 0.0011		Differential expression in GR vs. untreated; no direct GR vs. PR comparison
	miR-100-5p	Altered in IFNβ1a-treated patients (no acute change after injection)		*p* = 0.0002		Differential expression in GR vs. untreated; restored toward HC levels; no direct GR vs. PR comparison
	miR-3614	Increased 48 h after IFNβ1a injection		*p* = 0.0005		Acutely induced after IFNβ1a; not specifically associated with response status
	miR-107	N/A for treatment-induced change		N/R		No association with treatment response (associated with EDSS/disease severity)
Ebrahimkhani et al. 2020 [24]	miR-150-5p	↓ at 6 m in +R	Limma moderated *t*-test; logistic regression; Random Forest; ROC; Fisher’s exact test	*p* = 0.0012 (Bvs6m, +R)	N/R ^†^	Longitudinal change in +R; no direct R vs. NR test
	miR-548e-3p	↓ at 6 m in +R		*p* = 0.005 (Bvs6m, +R)		Longitudinal change in +R; no direct R vs. NR test
	miR-130b-3p	↑ at 6 m in +R		*p* = 0.004 (Bvs6m, +R)		Longitudinal change in +R; no direct R vs. NR test
	miR-654-5p	↑ at 6 m in +R		*p* = 0.047 (Bvs6m, +R)		Longitudinal change in +R; no direct R vs. NR test
	miR-487b-3p	↑ at 6 m in +R		*p* = 0.027 (Bvs6m, +R)		Longitudinal change in +R; no direct R vs. NR test
	miR-203a miR-193a-5p miR-379-5p miR-370-3p miR-382-5p miR-493-3 miR-432-5p miR-485-5p miR-2110 miR-1307-3p miR-1908-5p	↑ at 6 m in +R		*p* < 0.05 (Bvs6m, SR)		Longitudinal change in +R; no direct R vs. NR test
Giuliani et al. 2021 [25]	miR-34a-5p	ns change	Mann–Whitney U; Wilcoxon; Spearman’s correlation	Ns	N/P	No association reported
	miR-125a-5p	↓ after treatment		*p* = 0.028 ^•^ (progression), *p* = 0.001 **^■^** (B vs. 4 m)		Lower 4-month levels associated with disability progression
	miR-146a-5p	↓ after treatment		*p* = 0.042 ^•^ (progression), *p* < 0.001 **^■^** (B vs. 4 m)		Lower 4-month levels associated with disability progression
	miR-155	↓ after treatment		*p* = 0.013 **^■^** (B vs. 4 m)		No association reported

AUC, area under the receiver operating characteristic curve; B, baseline; GR, good responder; HC, healthy control; IFN, interferon; m, month; N/A, not assessed; N/R, not reported; N/P, not performed; NR, non-responder; ns, not significant; PR, poor responder; R, responder; SR, stable responder; +R, positive responder; ↓, downregulated; ↑, upregulated. ^•^ *p*-value reported for a Mann–Whitney *U* test. **^■^ ***p*-value reported for a Wilcoxon test. ^†^ AUC reported only for “active” vs. “quiescent” classification.

**Table 12:** Summary of the risk of bias assessment of the included studies.

Study Design	Tool Applied	Overall Risk of Bias	Notes
Prospective longitudinal studies (n = 7)	NOS-Cohort	moderate	Good selection and outcome assessment; limited comparability due to lack of confounder adjustment and absence of a comparator group
Case–control studies (n = 3)	NOS Case–Control	moderate	Adequate selection and exposure assessment; limited comparability with insufficient control for confounders
Cross-sectional studies (n = 5)	JBI Critical Appraisal Checklist	moderate	Clear inclusion criteria and measurement methods; no identification or control of confounding

JBI, Joanna Briggs Institute; NOS, Newcastle–Ottawa Scale.

## Data Availability

Data supporting the findings of this study are available within the article and its Supplementary Materials.

## Supplementary Materials

The following supporting information can be downloaded at https://www.mdpi.com/article/10.3390/ijms27146138/s1.

## Abbreviations

The following abbreviations are used in this manuscript:

MS	Multiple sclerosis
CNS	Central nervous system
BBB	Blood–brain barrier
DMTs	Disease-modifying therapies
MRI	Magnetic resonance imaging
NfLs	Neurofilaments
GFAP	Glial fibrillary acidic protein
CSF	Cerebrospinal fluid
miRNAs	MicroRNAs
RNA	Ribonucleic acid
RRMS	Relapsing–remitting multiple sclerosis
SPMS	Secondary progressive multiple sclerosis
PBMCs	Peripheral blood mononuclear cells
PBL	Peripheral blood leukocytes
EDSS	Expanded disability status scale
IFN-β	Interferon- β
DMF	Dimethyl fumarate
NEDA	No evidence of disease activity
EDA	Evidence of disease activity
CDP	Confirmed disability progression
HC	Healthy controls
GA	Glatiramer acetate
FTY720	Fingolimod
NTZ	Natalizumab
RT-qPCR	Reverse Transcription-quantitative Polymerase Chain Reaction
