# Supplementary material for: MicroRNA Expression Profiles as Biomarkers of Response to Disease-Modifying Therapies in Multiple Sclerosis: A Systematic Review
Source: Int J Mol Sci. 2026 Jul 9;27(14):6138. doi: 10.3390/ijms27146138 (PMC13410017; doi:10.3390/ijms27146138)
Supplement: Supplementary file 1 [file ijms-27-06138-s001.zip › PROSPERO registration protocol_final version.pdf]

NIHR | National Institute for Health and Care Research

PROSPERO
International prospective register of systematic reviews

MicroRNA Expression Profiles as Biomarkers of Response to Disease-Modifying Therapies in Multiple Sclerosis: A Systematic Review

Mihai Ioan Dumitreasa, Rodica Balasa, Smaranda Maier

Citation
Mihai Ioan Dumitreasa, Rodica Balasa, Smaranda Maier. MicroRNA Expression Profiles as Biomarkers of Response to Disease-Modifying Therapies in Multiple Sclerosis: A Systematic Review. PROSPERO 2026 CRD420261305887. Available from https://www.crd.york.ac.uk/PROSPERO/view/CRD420261305887.

REVIEW TITLE AND BASIC DETAILS

Review title

MicroRNA Expression Profiles as Biomarkers of Response to Disease-Modifying Therapies in Multiple Sclerosis: A Systematic Review

Condition or domain being studied

Multiple Sclerosis; Disease Modifying Drugs for Multiple Sclerosis; Biomarker analysis; Gene Expression

This review focuses on microRNA (miRNA) expression (including exosomal miRNAs) measured in blood-derived samples (plasma, serum, whole blood, PBMCs) and cerebrospinal fluid, as biomarkers of response to disease-modifying therapies in adults with multiple sclerosis.

Rationale for the review

Multiple sclerosis (MS) is a chronic, heterogeneous immune-mediated disease of the central nervous system. The remarkable progress in understanding the underlying pathogenic mechanisms has led to the development of disease-modifying therapies (DMTs), aimed at reducing inflammatory activity, relapse rates and slowing disease progression. However, the variability in treatment response represents a major challenge in clinical decision-making and highlights the need for reliable tools to evaluate, monitor and predict treatment response. In contrast to neurofilaments and glial fibrillary acidic protein, which become elevated as a response to tissue injury, miRNAs seem to be involved in the early phases of MS pathogenesis by regulating the

differentiation, maturation and function of T and B cells. Several studies investigated the dynamics of certain miRNAs, and observed not only that their expression is dysregulated in MS patients compared to healthy controls, but also that their expression is influenced by different DMTs. These findings suggest that miRNAs may serve as promising biomarkers of diagnosis, treatment responsiveness and monitoring, as well as potential therapeutic targets.

Despite the increasing volume of literature investigating miRNA profiles in MS, limitations regarding small sample sizes and heterogeneity in terms of miRNAs analyzed, study design, and patient population persist and prevents the validation of clinically applicable biomarkers. The aim of this systematic review is to evaluate the current research status regarding miRNA expression in adult patients with MS, in response to DMTs with a particular focus on differences between responders and non-responders and on longitudinal changes before and after treatment initiation.

Review objectives

To systematically review the evidence on blood- and cerebrospinal fluid–derived microRNA expression, including exosomal microRNAs, as biomarkers of response to disease-modifying therapies in adults with multiple sclerosis, and to evaluate treatment-related longitudinal changes in expression.

Keywords

Multiple sclerosis; Disease-modifying therapies; Biomarkers; MicroRNA

Country

Romania

ELIGIBILITY CRITERIA

Population

Included
Adults (≥18 years) diagnosed with relapsing–remitting multiple sclerosis (RRMS) according to the McDonald criteria or other established diagnostic criteria will be included.

Studies including mixed MS phenotypes (RRMS, secondary progressive MS, or primary progressive MS) will be eligible only if data for RRMS patients are reported separately or if the majority of participants have RRMS.

Intervention(s) or exposure(s)

Included
Disease Modifying Drugs for Multiple Sclerosis

Approved disease-modifying therapies for multiple sclerosis, including injectable, oral, and monoclonal antibody treatments.

Comparator(s) or control(s)

Included
PICO tags selected: Evaluating Response To Treatment; Biomarker analysis

Studies reporting (1) differences in microRNA expression between treatment responders and non-responders and/or (2) longitudinal changes in microRNA expression following initiation of a disease-modifying therapy, provided that these changes are evaluated in relation to clinical or radiological treatment response, will be eligible.

Study design

Only nonrandomized study types will be included.

Included

Observational studies (prospective or retrospective), including longitudinal and cross-sectional designs, will be eligible. Studies assessing differences in microRNA expression between treatment responders and non-responders and/or longitudinal changes before and after treatment initiation will be included.

Secondary analyses of clinical trials reporting microRNA expression data will also be considered eligible.

Excluded

In vitro studies, animal studies, case reports, reviews, editorials, and conference abstracts without full text will be excluded.

Context

Studies conducted in any healthcare setting (hospital-based or outpatient clinics) will be eligible. No restrictions will be applied regarding geographic location or healthcare system. The review will include studies evaluating microRNA expression in blood or cerebrospinal fluid samples obtained from adult patients with relapsing–remitting multiple sclerosis treated with approved disease-modifying therapies.

TIMELINE OF THE REVIEW

Date of first submission to PROSPERO

10 February 2026

Review timeline

Start date: 11 February 2026. End date: 15 May 2026.

Date of registration in PROSPERO

12 February 2026

AVAILABILITY OF FULL PROTOCOL

Availability of full protocol

A full protocol has been written and uploaded to PROSPERO. The protocol will be made available after the review is completed.

SEARCHING AND SCREENING

Search for unpublished studies
Only published studies will be sought.

Main sources that will be searched
The main databases to be searched are *MEDLINE*, *PubMed* and *Scopus*.

Search language restrictions
The review will only include studies published in English.

Search date restrictions
There are no search date restrictions.

Other methods of identifying studies
Other studies will be identified by: *looking through all the articles that cite the papers included in the review ("snowballing" or forward citation searching)* and *reference list checking (backward citation searching)*.

Link to search strategy
A full search strategy is available in the full protocol as described in the *Availability of full protocol* section

Selection process
Studies will be screened independently by at least two people (or person/machine combination) with a process to resolve differences.

Other relevant information about searching and screening
None

DATA COLLECTION PROCESS

Data extraction from published articles and reports
Data will be extracted independently by at least two people (or person/machine combination) with a process to resolve differences.

Authors will be asked to provide any required data not available in published reports.

Study risk of bias or quality assessment
Risk of bias will be assessed using: *Newcastle-Ottawa*

Data will be assessed independently by at least two people (or person/machine combination) with a process to resolve differences.

Additional information will be sought from study investigators if required information is unclear or unavailable in the study publications/reports.

Reporting bias assessment
Risk of bias due to missing results will be assessed

Certainty assessment
Certainty of findings will not be assessed

OUTCOMES TO BE ANALYSED

Main outcomes

Association between microRNA expression levels and treatment response, as defined by clinical and/or radiological criteria (e.g., relapse rate, disability progression, MRI activity).

Additional outcomes

Longitudinal changes in microRNA expression following initiation of disease-modifying therapies, when evaluated in relation to clinical and/or radiological treatment response.

PLANNED DATA SYNTHESIS

Strategy for data synthesis

A narrative synthesis of the included studies will be performed. Due to anticipated heterogeneity in study design, patient populations, definitions of treatment response, analyzed microRNAs, microRNA profiling methods, and biological sample types, a quantitative meta-analysis is not expected to be feasible.

Results will be summarized descriptively and organized according to type of disease-modifying therapy, sample type, specific microRNAs analyzed, and reported associations with treatment response. The direction of microRNA expression changes (upregulation or downregulation) and statistical significance will be presented in summary tables.

Clinical and methodological heterogeneity across studies will be evaluated qualitatively and discussed in the interpretation of findings. Risk of bias and study quality assessments will be considered in the interpretation of findings. If a subset of studies is found to be sufficiently homogeneous in terms of design, population, and outcome definition, a quantitative synthesis may be considered.

CURRENT REVIEW STAGE

Stage of the review at this submission 1 change

Review stage	Started	Completed
Pilot work	✓	✓
Formal searching/study identification	✓	✓
Screening search results against inclusion criteria	✓	✓
Data extraction or receipt of IPD	✓	✓
Risk of bias/quality assessment	✓	✓
Data synthesis	✓	✓

Review status

The review is completed.

Publication of review results
Results of the review will be published.

REVIEW AFFILIATION, FUNDING AND PEER REVIEW

Review team members
Mr Mihai Ioan Dumitreasa (review guarantor and contact) ORCID: 0009-0001-4587-7995. University of Medicine, Pharmacy, Sciences and Technology "George Emil Palade" of Târgu Mureş. Romania.

No conflict of interest declared.

Professor Rodica Balasa. University of Medicine Pharmacy Science and Technology "George Emil Palade" of Targu Mures. Romania.

No conflict of interest declared.

Associate Professor Smaranda Maier. University of Medicine, Pharmacy, Sciences and Technology "George Emil Palade" of Târgu Mureş. Romania.

No conflict of interest declared.

Named contact
Mr Mihai Ioan Dumitreasa (mihai.du96@gmail.com). ORCID: 0009-0001-4587-7995. University of Medicine, Pharmacy, Sciences and Technology "George Emil Palade" of Târgu Mureş. Romania.

Review affiliation
Doctoral School, "George Emil Palade" University of Medicine, Pharmacy, Science, and Technology of Targu Mures, 540142 Targu Mures, Romania.

Funding source
Review has no specific/external funding but is supported by guarantor/review team (non-commercial) institutions.

Peer review
There has been no peer review of this planned review.

ADDITIONAL INFORMATION

Review conflict of interest
Declared individual interests are recorded under team member details.. No additional interests are recorded for this review.

Medical Subject Headings
Multiple Sclerosis; Humans; Adult; Antibodies, Monoclonal; Biomarkers; MicroRNAs; Cerebrospinal Fluid; Classification

Revision note 1 change
Stage of the review was updated

SIMILAR REVIEWS

Check for similar records already in PROSPERO

PROSPERO identified a number of existing PROSPERO records that were similar to this one (last check made on 10 February 2026). These are shown below along with the reasons given by that the review team for the reviews being different and/or proceeding.

- Biological disease-modifying therapies for multiple sclerosis: a systematic review of evidence derived from phase III clinical trials [published 9 February 2026] [CRD420261305103]. The review was judged **not to be similar**
- Network meta-analysis investigating how well efficacy results from disease-modifying therapies in randomized controlled trials translate to effectiveness data in observational studies for patients with relapsing-remitting multiple sclerosis. [published 28 August 2022] [CRD42022354152]. The review was judged **not to be similar**
- Decision-Making Experiences of Patients with Multiple Sclerosis in Disease-Modifying Therapy: A Qualitative Meta-Synthesis [published 18 January 2026] [CRD420261283544]. The review was judged **not to be similar**

PROSPERO version history 1 change

- Version 1.2, published 21 Jun 2026
- Version 1.1, published 01 May 2026
- Version 1.0, published 12 Feb 2026

Disclaimer

The content of this record displays the information provided by the review team. PROSPERO does not peer review registration records or endorse their content.

PROSPERO accepts and posts the information provided in good faith; responsibility for record content rests with the review team. The guarantor for this record has affirmed that the information provided is truthful and that they understand that deliberate provision of inaccurate information may be construed as scientific misconduct.

PROSPERO does not accept any liability for the content provided in this record or for its use. Readers use the information provided in this record at their own risk.

Any enquiries about the record should be referred to the named review contact
