# Supplementary material for: MicroRNA Expression Profiles as Biomarkers of Response to Disease-Modifying Therapies in Multiple Sclerosis: A Systematic Review
Source: Int J Mol Sci. 2026 Jul 9;27(14):6138. doi: 10.3390/ijms27146138 (PMC13410017; doi:10.3390/ijms27146138)
Supplement: Supplementary file 1 [file ijms-27-06138-s001.zip › Search Strategy_supplementary material.pdf]

Supplementary Material – Detailed Search Strategies

PubMed → 79 results (08.02.2026)

("multiple sclerosis"[MeSH Terms] OR "multiple sclerosis"[Title/Abstract] OR MS[Title/Abstract]) AND

("MicroRNAs"[MeSH Terms] OR microRNA*[Title/Abstract] OR miRNA*[Title/Abstract] OR exosomal microRNA*[Title/Abstract] OR exosomal miRNA*[Title/Abstract] OR exomiR*[Title/Abstract]) AND

("disease-modifying therapy"[Title/Abstract] OR "disease modifying therapy"[Title/Abstract] OR DMT[Title/Abstract]

OR "interferon beta"[MeSH Terms] OR "interferon beta"[Title/Abstract] OR Avonex[Title/Abstract] OR Betaferon[Title/Abstract] OR Rebif[Title/Abstract]

OR "Glatiramer Acetate"[MeSH Terms] OR "glatiramer acetate"[Title/Abstract] OR Copaxone[Title/Abstract]

OR "dimethyl fumarate"[MeSH Terms] OR "dimethyl fumarate"[Title/Abstract] OR Tecfidera[Title/Abstract]

OR teriflunomide[Title/Abstract] OR Aubagio[Title/Abstract]

OR "Fingolimod hydrochloride"[MeSH Terms] OR fingolimod[Title/Abstract] OR FTY720[Title/Abstract] OR Gilenya[Title/Abstract]

OR siponimod[Title/Abstract] OR Mayzent[Title/Abstract]

OR ozanimod[Title/Abstract] OR Zeposia[Title/Abstract]

OR ponesimod[Title/Abstract] OR Ponvory[Title/Abstract]

OR "sphingosine 1 phosphate receptor modulators"[MeSH Terms] OR "sphingosine 1 phosphate receptor modulators"[Title/Abstract] OR S1P[Title/Abstract] OR "S1P receptor"[Title/Abstract]

OR "natalizumab"[MeSH Terms] OR natalizumab[Title/Abstract] OR Tysabri[Title/Abstract]

OR ocrelizumab[Title/Abstract] OR Ocrevus[Title/Abstract]

OR ofatumumab[Title/Abstract] OR Kesimpta[Title/Abstract]

OR "cladribine"[MeSH Terms] OR cladribine[Title/Abstract] OR Mavenclad[Title/Abstract]

OR "alemtuzumab"[MeSH Terms] OR alemtuzumab[Title/Abstract] OR Lemtrada[Title/Abstract]

OR ublituximab[Title/Abstract] OR Briumvi[Title/Abstract]

OR mitoxantrone[Title/Abstract]

OR diroximel fumarate[Title/Abstract] OR Vumerity[Title/Abstract])

WebOfScience → 134 results (16.02.2026)

TS=("multiple sclerosis" OR MS)

AND TS=(microRNA* OR miRNA* OR "exosomal microRNA*" OR "exosomal miRNA*" OR exomiR*)

AND TS=("disease-modifying therapy" OR "disease modifying therapy" OR DMT

OR "interferon beta" OR Avonex OR Betaferon OR Rebif

OR "glatiramer acetate" OR Copaxone

OR "dimethyl fumarate" OR Tecfidera

OR teriflunomide OR Aubagio

OR fingolimod OR FTY720 OR Gilenya

OR siponimod OR Mayzent

OR ozanimod OR Zeposia

OR ponesimod OR Ponvory

OR "sphingosine 1 phosphate receptor modulators" OR "S1P receptor"

OR natalizumab OR Tysabri

OR ocrelizumab OR Ocrevus

OR ofatumumab OR Kesimpta

OR cladribine OR Mavenclad

OR alemtuzumab OR Lemtrada

OR ublituximab OR Briumvi

OR mitoxantrone

OR diroximel fumarate OR Vumerity)
